# Pericyte NF-*κ*B activation enhances endothelial cell proliferation and proangiogenic cytokine secretion in vitro

**DOI:** 10.14814/phy2.12309

**Published:** 2015-04-27

**Authors:** Katherine E LaBarbera, Robert D Hyldahl, Kevin S O'Fallon, Priscilla M Clarkson, Sarah Witkowski

**Affiliations:** 1 Department of Kinesiology, University of Massachusetts AmherstAmherst, Massachusetts; 2 Department of Exercise Sciences, Brigham Young UniversityProvo, Utah

**Keywords:** Cytokine signaling, microvasculature, NF-*κ*B, pericyte

## Abstract

Pericytes are skeletal muscle resident, multipotent stem cells that are localized to the microvasculature. In vivo, studies have shown that they respond to damage through activation of nuclear-factor kappa-B (NF-*κ*B), but the downstream effects of NF-*κ*B activation on endothelial cell proliferation and cell–cell signaling during repair remain unknown. The purpose of this study was to examine pericyte NF-*κ*B activation in a model of skeletal muscle damage; and use genetic manipulation to study the effects of changes in pericyte NF-*κ*B activation on endothelial cell proliferation and cytokine secretion. We utilized scratch injury to C_2_C_12_ cells in coculture with human primary pericytes to assess NF-*κ*B activation and monocyte chemoattractant protein-1 (MCP-1) secretion from pericytes and C_2_C_12_ cells. We also cocultured endothelial cells with pericytes that expressed genetically altered NF-*κ*B activation levels, and then quantified endothelial cell proliferation and screened the conditioned media for secreted cytokines. Pericytes trended toward greater NF-*κ*B activation in injured compared to control cocultures (*P* = 0.085) and in comparison to C_2_C_12_ cells (*P* = 0.079). Second, increased NF-*κ*B activation in pericytes enhanced the proliferation of cocultured endothelial cells (1.3-fold, *P* = 0.002). Finally, we identified inflammatory signaling molecules, including MCP-1 and interleukin 8 (IL-8) that may mediate the crosstalk between pericytes and endothelial cells. The results of this study show that pericyte NF-*κ*B activation may be an important mechanism in skeletal muscle repair with implications for the development of therapies for musculoskeletal and vascular diseases, including peripheral artery disease.

## Introduction

Skeletal muscle damage following injury, disease, or unaccustomed exercise stimulates coordinated repair processes from muscle and nonmuscle cell types, including cells associated with the vasculature (Shi and Garry 2006; Hyldahl et al. 2011; Boppart et al. 2013). Skeletal muscle is a highly vascularized tissue with reports that there are as many as 4–5 capillaries in contact with each muscle fiber in the vastus lateralis of young healthy males (Groen et al. 2014). Capillaries and other microvascular vessels, including precapillary arterioles and postcapillary venules, have a single layer of endothelial cells on the luminal surface. There is evidence that skeletal muscle-damaging exercise affects these endothelial cells and causes microvascular dysfunction (Kano et al. 2004, 2005). Additionally, pericytes, which are cells found on the abluminal surface of endothelial cells throughout the microvasculature, may contribute to skeletal muscle repair (Dellavalle et al. 2011; Hyldahl et al. 2011). The consequences of skeletal muscle damage on the microvasculature, including angiogenic processes or endothelial cell crosstalk with pericytes remain unknown.

Pericytes are tissue-resident multipotent stem cells that are ubiquitous throughout the microvasculature where they reside under the basement membrane at a density ranging between 1:10 and 1:100 pericytes to endothelial cells within skeletal muscle tissue (Tilton et al. 1979; Shepro and Morel 1993; Hirschi and D'Amore 1996). They are mesenchymal-like stem cells (Crisan et al. 2008) that have the potential to differentiate down mesodermal lineages toward adipogenic (Richardson et al. 1982), osteogenic (Diaz-Flores et al. 1992), and myogenic fates (Dellavalle et al. 2011). Reports indicate that pericytes can fuse with muscle fibers and contribute to the satellite cell pool in vivo (Dellavalle et al. 2011). Additionally, pericytes may contribute to skeletal muscle repair (Sassoli et al. 2012) and cardiac muscle repair (Chen et al. 2013) through paracrine effects, although the signaling molecules remain largely unidentified. Previous work by Hyldahl et al. (2011) has highlighted pericyte NF-*κ*B activity as a potential mechanism for pericyte involvement in muscle repair. They showed that a muscle-damaging eccentric exercise in humans caused NF-*κ*B activation in muscle-resident pericytes 3 h after exercise of the quadriceps (Hyldahl et al. 2011). In a follow up in vitro study, it was shown that pericyte NF-*κ*B activation enhanced skeletal myoblast proliferation and inhibited myoblast differentiation (Hyldahl et al. 2013). The signals downstream of NF-*κ*B that mediate the cellular crosstalk between pericytes and muscle cells have not been investigated.

Only a few studies have investigated the complex relationship between endothelial cells, pericytes, and skeletal muscle following tissue damage. In a severe model of muscle injury, Tamaki et al. (2005) showed that muscle-derived mesenchymal stem cells (CD34^+^/CD45^−^), which are similar to pericytes in anatomical location, cell surface marker expression, and function (Covas et al. 2008), contributed to both vasculogenesis and myogenesis during regeneration. Huntsman et al. (2013) reported that pericytes contributed to arteriogenesis following 4 weeks of eccentric exercise training in a transgenic animal model. This same group investigated the potential paracrine signaling factors secreted by pericytes in response to in vitro mechanical strain. They identified epidermal growth factor (EGF), vascular endothelial growth factor (VEGF), and interferon gamma-induced protein 10 (IP-10) as potential mediators of the arteriogenic response (Huntsman et al. 2013). IP-10 is a target of NF-*κ*B, which supports the hypothesis that NF-*κ*B activation in pericytes may be an important mediator of cellular crosstalk with muscle and endothelial cells by regulating the transcription of inflammatory cytokines. Another gene target of NF-*κ*B is monocyte chemoattractant protein-1 (MCP-1). Evidence from both in vitro (Peterson and Pizza 2009) and in vivo studies (Chen et al. 2003; Hyldahl et al. 2011) implicates MCP-1 as an important cytokine in muscle repair, where its main role is the chemoattraction of leukocytes, but may also have roles in angiogenesis and regeneration (Yahiaoui et al. 2008; Lu et al. 2011). Other proinflammatory cytokines known to be under the transcriptional regulation of NF-*κ*B, such as granulocyte macrophage-colony stimulating factor (GM-CSF), may also be secreted from pericytes with potential downstream effects on endothelial cells (Huntsman et al. 2013).

Due to the close physical proximity between pericytes and endothelial cells, as well as their interdependence for maintaining microvascular homeostasis, we have hypothesized that pericyte NF-*κ*B activation following muscle damage may also influence microvascular endothelial cells. While evidence suggests that pericyte NF-*κ*B activation following muscle damage may enhance myoblast proliferation, little is known about the effects of pericyte NF-*κ*B activation on endothelial cell proliferation and cell–cell signaling. Therefore, the purpose of this study was to examine pericyte NF-*κ*B activation in a model of skeletal muscle damage; and use genetic manipulation to study how pericyte NF-*κ*B activation affects endothelial cell proliferation and cytokine secretion.

## Materials and Methods

### Cell culture

Human primary pericytes isolated from placental tissue were purchased from PromoCell (Heidelberg, Germany). As reported by the supplier, the pericytes were positive for the mesenchymal stem cell markers CD146 and CD105 and negative for the endothelial cell markers CD31 and CD34. Immunofluorescence experiments confirmed that the cells also expressed the pericyte marker NG2 (data not shown). The cells displayed a stellate morphology characteristic of pericytes (Bryan and D'Amore 2008). Pericytes between passages 6 and 9 were used for experiments. Cultures were maintained at 37°C in a 5% CO_2_ incubator in growth medium consisting of DMEM supplemented with 10% FBS and 1% penicillin and streptomycin. Cultures were passaged using 0.25% trypsin, 0.1% EDTA before reaching 80% confluence. C_2_C_12_ myoblasts were obtained from the American Type Culture Collection (ATCC, Manassas, VA) and cultured under the same conditions as the pericytes. To obtain myotube cultures, C_2_C_12_ myoblasts were cultured in reduced growth factor medium containing DMEM supplemented with 2% horse serum and 1% penicillin and streptomycin for 4 days to induce differentiation into mature myotube cultures. Human microvascular endothelial cells (HMVECs) are an hTERT immortalized cell line (Shao and Guo 2004) that was cultured in endothelial basal medium-2 (EBM-2; Lonza, Walkersville, MD) supplemented with 10% FBS, 1% penicillin and streptomycin, 1 *μ*g/mL EGF, and 50 *μ*g/mL hydrocortisone.

### Pericyte/C_2_C_12_ coculture

A transwell coculture system was used to facilitate exchange of soluble factors while preventing the migration of cells between wells (0.4 *μ*m pore size, Corning Life Sciences, Tewksbury, MA). C_2_C_12_ myotube cultures were grown in a 6-well plate format with pericytes seeded in the transwell insert. Cocultures were maintained in a 5% CO_2_ incubator and culture medium was replaced every 24 h at 4 days post myotube differentiation, which coincided with ∽80% pericyte confluency, culture medium was replaced 3 h prior to the start of the experiment to allow cells to equilibrate to fresh medium. Next, myotube cultures were scratch-injured, which created an open “wound” on the culture plate. To impart the scratch-injury, a sterile gel-loading pipette tip was used to generate a continuous and well-delineated wound region along the surface of each plate producing a total wound area equivalent to ∽10% of the total surface area of the C_2_C_12_ cell monolayer, similarly to the methods previously described where scrape injury was shown to cause myotube damage through lactate dehydrogenase release (Tsivitse et al. 2005). In parallel, control cocultures were established where no injury was imparted to C_2_C_12_ cells. Three replicates were performed for a baseline (BSLN), 3, 12, and 24 h time point for uninjured control (CON) and injured (INJ) cultures. At each time point, cell culture supernatant was collected and immediately frozen at −80°C for subsequent analysis of secreted MCP-1. Also at each time point, nuclear extracts from pericytes and C_2_C_12_ cells, respectively, were isolated via differential centrifugation using a cellular extraction kit according to manufacturer's instructions (Nuclear Extract Kit; Active Motif, Carlsbad, CA) and immediately frozen at −80°C.

### ELISA-based NF-κB activation

Total nuclear protein content was determined using a BSA-based protein quantification assay (ProStain; Active Motif). An ELISA-based transcription factor assay kit (TransAM NF-*κ*B p65 Assay Kit; Active Motif) was used to quantify NF-*κ*B p65 subunit nuclear DNA binding activity in pericytes and C_2_C_12_ cells from control and injured cocultures according to manufacturer's instructions. Briefly, 2 *μ*g of nuclear protein was added to wells coated with a consensus binding sequence for NF-*κ*B (5′-GGGACTTTCC-3′) and incubated for 1 h at room temperature. Wild type and mutated consensus oligonucleotides were used as competitors for NF-*κ*B binding to ensure specificity of the reaction as per manufacturer's instructions. Wells were then washed, and a primary antibody directed at the p65 subunit was added and left to incubate for 1 h. This was followed by treatment of all wells with a secondary horseradish peroxidase-conjugated antibody. Then, developing solution was added to initiate a colorimetric reaction. After 5 min, a stop solution was added and absorbance was measured at 450 nm on a multiwell microplate reader (FLUOstar Optima; BMG Labtech, Offenburg, Germany). All samples were assayed in duplicate, and averages were used for data analysis.

### MCP-1 secretion

To determine MCP-1 secretion from human pericytes and murine C_2_C_12_ cells, respective ELISAs for the detection of human (Human CCL2/MCP-1 Quantikine ELISA kit; R&D Systems, Minneapolis, MN) and murine MCP-1 (Mouse /Rat CCL2/JE/MCP-1 Quantikine ELISA kit; R&D Systems) were performed on cell culture supernatants. The kits were tested for species cross reactivity by the manufacturer, and no cross reactivity was detected. Assay kits employed the quantitative sandwich ELISA technique, and were performed on undiluted samples according to manufacturer's instructions. A multiwell microplate reader was used (FLUOstar Optima; BMG Labtech) to measure absorbance at 450 nm. Assays were performed in duplicate and average values were used for analysis.

### Pericyte/HMVEC coculture

Pericytes were seeded in triplicate in a 6-well plate format following transient transfection (described below) with vectors designed to alter NF-*κ*B activity. At 24 h after transfection, medium was replaced with fresh medium and coculture was initiated by seeding HMVECs onto transwell inserts (0.4 *μ*m pore size, Corning Life Sciences, Tewksbury, MA). At 24 and 48 h after the initiation of coculture, cell culture supernatants were collected and immediately frozen at −80°C for analysis of cytokine secretion. Endothelial cell number was quantified at 24 and 48 h (detailed below).

### Transient transfections

Expression plasmids were used to alter NF-*κ*B activation in pericytes. A dominant negative (d.n.) IKK*β* (K44M) was used to decrease NF-*κ*B activation. It encodes a kinase dead form of IKK*β*, and was developed in the laboratory of Michael Karin, PhD (University of California, San Diego, CA). A constitutively active (c.a.) IKK*β* (S177/S188→EE) was used to increase NF-*κ*B activation by encoding for a constitutively active form of IKK*β*, and was developed in the lab of Steven Shoelson, MD PhD (Joslin Diabetes Center, Boston, MA). For a control that did not alter NF-*κ*B activation, an empty vector of the expression plasmid was used (pEF6/HisB; Invitrogen, Carlsbad, CA). For visualization of the transfected proteins, the d.n. IKK*β* and c.a. IKK*β* were subcloned into the N-terminus of an EGFP expression vector (pEGFP-n1; Clontech, Palo Alto, CA) by the laboratory of Susan C Kandarian, PhD (Boston University, Boston, MA) to create the d.n. IKK*β*-EGFP and c.a. IKK*β*-EGFP fusion proteins. A Basic Nucleofector Kit for Primary Smooth Muscle Cells (Lonza, Walkersville, MD) and a Nucleofector II machine (Amaxa, Gaithersburg, MD) using the P-013 program was used to electroporate 5 *μ*g of each experimental plasmid plus 5 *μ*g of the pNF-kB-MetLuc2-Reporter plasmid (Clontech, Mountain View, CA) into 5 × 10^5^ pericytes. Following electroporation of each respective condition, pericytes were seeded in triplicate in a 6-well plate.

### Luciferase reporter assay

At 24 h post transfection, culture medium was collected from each condition and assayed for luciferase using a Ready-to-Glow Secreted Luciferase Reporter Assay (Clontech, Mountain View, CA) according to manufacturer's instructions. The assay was run in triplicate and luminescence was measured on a FLUOstar Optima plate reader (BMG Labtech).

### Endothelial cell number

At 24 and 48 h after coculture initiation, endothelial cells were harvested from the transwell membrane using 0.25% trypsin, 0.1% EDTA, and then cell number was quantified using Trypan Blue staining and a hemocytometer. Two cell counts were performed for each of the three replicates per condition. Averages of the two counts were used for statistical analysis.

### Multiplex cytokine secretion assessment

Cell culture supernatant was collected from pericyte/HMVEC cocultures at 24 h after coculture initiation. Cell culture supernatant was also collected from pericyte monocultures at the same time point. Supernatant was centrifuged at 4°C for 10 min at 1500 rpm. Protein was quantified using ProStain Protein Quantification kit (Active Motif), and equal amounts of protein were assayed for cytokine and chemokine concentration using a Luminex Magpix multiplexing platform (Luminex Corporation, Austin, TX). Quantification of cytokine/chemokine concentrations in the culture supernatants was carried out with a human cytokine/chemokine 42-plex bead panel (Millipore Corporation, Billerica, MA). Mutiplexing analysis was performed using reagents supplied by Millipore according to the manufacturers recommendations. Briefly, antibody-conjugated magnetic beads were incubated with cell culture supernatants (antigen) in a 96-well format followed by sequential incubations with biotinylated detection antibody and streptavidin-phycoerythrin. Bead complexes were then read on the Magpix multiplex platform (Luminex Corporation). Sensitivity of standards ranged between 3.2 and 10,000 pg/mL, giving a broad range of sensitivity. Standard curves and data analysis was performed using Milliplex Analyst 5.1 software (Millipore Corporation).

### Statistical analysis

A 3-way ANOVA was used to determine differences in NF-*κ*B activation for the main effects of cell type (pericytes versus C_2_C_12_), time (BSLN, 3, 6, 24 h), and condition (control versus injured). Two-way ANOVA was used to investigate differences in NF-*κ*B activation of respective cells types over time (BSLN, 3, 6, 24 h) and between conditions (control versus injured). A 2-way ANOVA was used to determine differences in endothelial cell proliferation between conditions (c.a. IKK*β*, d.n. IKK*β*, and e.v) and over time (24 and 48 h). Multiplex cytokine data for each individual cytokine of interest were tested for differences among conditions using a 1-way ANOVA. Significant main effects and interactions were investigated using *t*-tests or Tukey's honest significant difference post hoc test where appropriate. Significance was set a priori at *P* < 0.05.

## Results

### NF-κB activation in an in vitro pericyte/C_2_C_12_ coculture model of muscle injury

**Figure 1:**
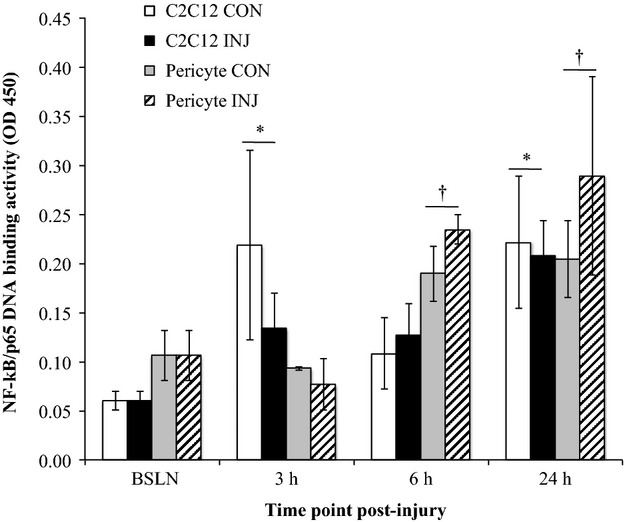
Time course of NF-*κ*B activation in cocultured C_2_C_12_ cells and pericytes. p65 DNA binding activity for cocultured C_2_C_12_ cells and pericytes in uninjured control (CON) and scratch-injured (INJ) conditions at baseline (BSLN), 3, 6, and 24 h time points. Data are means ± SD. *Significantly increased compared to BSLN for C_2_C_12_ cells. ^†^Significantly increased compared to BSLN for pericytes.

Pericytes were cocultured with C_2_C_12_ myotubes using transwell inserts to examine the time course of pericyte and muscle cell NF-*κ*B activation. In this coculture model, the C_2_C_12_ p65 DNA binding activity was significantly elevated at 3 h (2.5-fold, *P* = 0.027) and 24 h (3.57-fold, *P* = 0.001) relative to BSLN. There was no difference in C_2_C_12_ p65 DNA binding activity in INJ compared to CON (*P* = 0.698). In the same cocultures, pericyte p65 DNA binding activity was increased relative to BSLN at 6 h (2.0-fold, *P* = 0.007), and 24 h (2.33-fold, *P* = 0.001). Pericytes trended toward greater p65 DNA binding activity in INJ compared to CON (*P* = 0.085). Further, pericytes trended toward greater overall p65 DNA binding activity compared to C_2_C_12_ cells (*P* = 0.079) (Fig.1).

### MCP-1 is secreted by pericytes in pericyte/C_2_C_12_ coculture model of muscle injury

**Figure 2:**
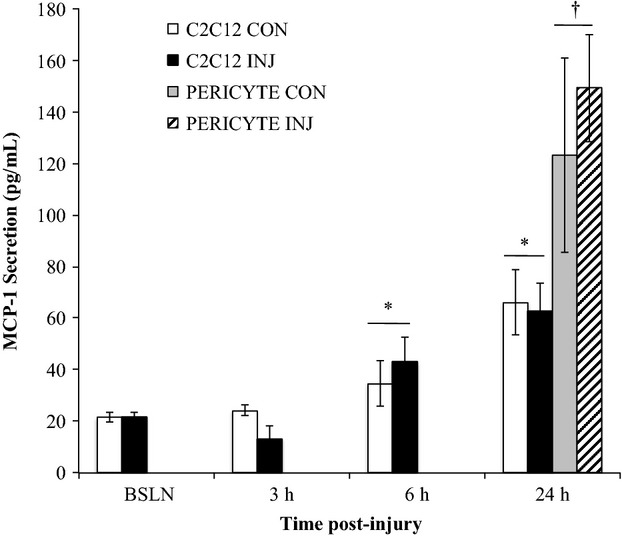
Time course of MCP-1 secretion from cocultured pericytes and C_2_C_12_ cells. Monocyte chemoattractant protein-1 (MCP-1) secretion by cocultured C_2_C_12_ cells and pericytes in uninjured control (CON) and scratch-injured (INJ) conditions at baseline (BSLN), 3, 6, and 24 h time points. Data are means ± SD. *Significantly increased compared to BSLN for C_2_C_12_ cells. ^†^Significantly greater vs. C_2_C_12_ cells at 24 h.

The pericyte and muscle cell coculture model was utilized to investigate secreted signaling molecules involved in pericyte-muscle cell crosstalk. In the coculture model, pericyte MCP-1 secretion was first detected 24 h post injury, and it exceeded C_2_C_12_ MCP-1 secretion (2.1-fold, *P* < 0.001) at this time point in INJ and CON conditions. C_2_C_12_ MCP-1 secretion was increased compared to BSLN at 6 h (1.6-fold, *P* = 0.032) and 24 h post injury (3.0-fold, *P* < 0.001). There were no differences in MCP-1 secretion between INJ and CON for pericytes or C_2_C_12_ cells (Fig.2).

### Pericyte NF-κB genetic manipulation

**Figure 3:**
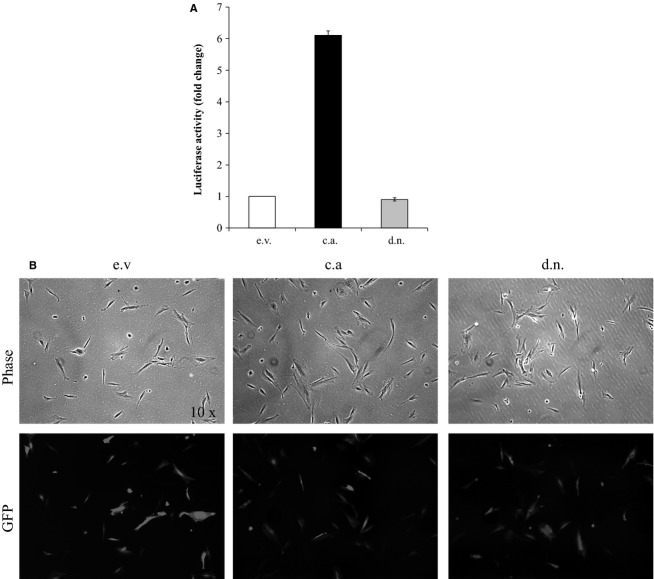
Pericyte transfection efficacy and efficiency in altering NF-*κ*B activation. (A) Representative images of pericytes transfected with vectors designed to enhance (constitutively active (c.a.) IKK*β*-EGFP), diminish (dominant negative (d.n.) IKK*β*-EGFP), or have no effect on (empty vector (e.v.) pEF6/HisB) NF-*κ*B activity at 24 h post transfection. (B) A luciferase reporter system was used to assess the ability of expression plasmids to alter NF-*κ*B expression. Data are means ± SD.

To investigate the downstream effects of pericyte NF-*κ*B activation, an in vitro coculture model was developed that utilized pericytes with genetically altered NF-*κ*B activation. Pericytes were genetically altered via transfection with expression plasmids designed to enhance (c.a. IKK*β*-EGFP), reduce (d.n. IKK*β*-EGFP), or have no impact (e.v. EGFP) on NF-*κ*B activity. The transfection efficiency was approximately 65–75% based on the percentage of EGFP fluorescent pericytes at 24 h after transfection (Fig.3A). Transfection efficiency was not different between conditions. The transfection efficacy was assessed using a luciferase assay (Fig.3B). At 24 h post transfection, luciferase activity was increased 6.1-fold in the c.a. IKK*β* condition compared to the e.v. control condition. The luciferase activity in the d.n. IKK*β* was decreased 10% compared to e.v. control condition.

### Pericyte NF-κB activation enhances HMVEC proliferation in coculture

**Figure 4:**
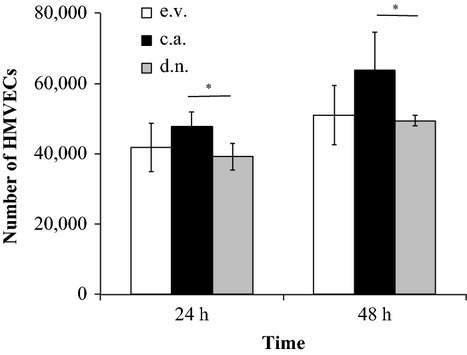
HMVEC proliferation is affected by pericyte NF-*κ*B activation in coculture. Human microvascular endothelial cell (HMVEC) number in coculture with pericytes expressing constitutively active IKK*β* (c.a.), dominant negative IKK*β* (d.n.), or empty vector control (e.v.). Data are means ± SD. *Significant difference between c.a. and d.n.

Genetically altered pericytes were cocultured with HMVECs using transwell inserts to investigate the effects of altered pericyte NF-*κ*B activation on endothelial cell proliferation. In pericyte/HMVEC cocultures, there was an increase in HMVEC cell number, a measure of proliferation, from 24 to 48 h for all conditions (*P* = 0.002, 1.3-fold). HMVEC proliferation was significantly greater in the c.a. IKK*β* pericyte coculture condition compared to the d.n. IKK*β* pericyte coculture condition (*P* = 0.002, 1.3-fold; Fig.4).

### Cytokine secretion from pericyte monocultures and pericyte/HMVEC cocultures

To assess signaling molecules that may mediate the proliferative response in endothelial cells, cell culture supernatant was assayed for cytokine concentration. Cell culture supernatants were collected in pericyte/HMVEC cocultures at 24 h after the initiation of coculture and at the corresponding time point in pericyte monocultures.

The following cytokines were secreted from pericytes in monoculture in the c.a. IKK*β* condition, but were not detected in the d.n. IKK*β* condition or e.v. control condition: eotaxin (15.61 ± 2.7 *ρ*g/mL), granulocyte colony-stimulating factor (G-CSF, 17.21 ± 4.6 *ρ*g/mL), GM-CSF (15.16 ± 4.6 *ρ*g/mL), fractalkine (CX3CL1, 30.87 ± 5.3 *ρ*g/mL), interferon alpha-2 (IFNA2, 8.62 ± 1.6 *ρ*g/mL), growth-regulated oncogene (GRO, 8.41 ± 6.0 *ρ*g/mL), interleukin 6 (IL-6, 45.52 ± 9.7 *ρ*g/mL), interleukin 7 (IL-7, 11.27 ± 2.0 *ρ*g/mL), IP-10 (196.56 ± 91.3 *ρ*g/mL), and macrophage inflammatory protein-1 alpha (MIP-1*α*, 38.57 ± 10.9 *ρ*g/mL). There was greater cytokine secretion in the c.a. IKK*β* condition compared to both d.n. IKK*β* (*P* < 0.01) and e.v. conditions (*P* < 0.01), respectively, for the following cytokines: interleukin 8 (IL-8, 599.16 ± 192.5 vs. 1.70 ± 0.9 and 3.54 ± 1.1 *ρ*g/mL), MCP-1 (32.56 ± 9.3 vs. 4.14 ± 1.7 and 5.00 ± 2.6 *ρ*g/mL), and regulated on activation, normal T-cell expressed and secreted (RANTES, 209.18 ± 73.3 vs. 4.24 ± 2.0 and 5.96 ± 3.1 *ρ*g/mL).

**Figure 5:**
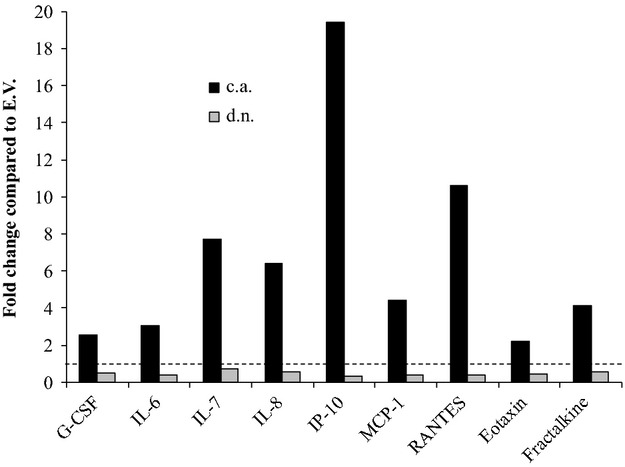
Pericyte NF-*κ*B activation affects cytokine secretion in pericyte/HMVEC cocultures. Cytokine secretion of granulocyte-colony stimulating factor (G-CSF), fractalkine, interleukin 6 (IL-6), interleukin 7 (IL-7), interleukin 8 (IL-8), interferon gamma-induced protein-10 (IP-10), monocyte chemoattractant protein-1 (MCP-1), regulated on activation, normal T-cell expressed and secreted (RANTES), and eotaxin in the cocultures of human microvascular endothelial cells (HMVECs) and pericytes that were genetically altered to express constitutively active IKK*β* (c.a.) or dominant negative IKK*β* (d.n.) in comparison to empty vector control (e.v.). Significantly greater cytokine concentration was observed in the c.a. IKK*β* condition compared to both e.v. and d.n. IKK*β* conditions for all cytokines (*P* < 0.05).

In cocultures, there was greater cytokine secretion in the c.a. IKK*β* condition compared to both d.n. IKK*β* (*P* < 0.05) and e.v. conditions (*P* < 0.05), respectively, for the following cytokines: eotaxin (16.16 ± 0.6 vs. 3.32 ± 4.7 and 7.26 ± 1.3 *ρ*g/mL), G-CSF (55.81 ± 11.6 vs. 10.27 ± 5.4 and 22.13 ± 4.8 *ρ*g/mL), RANTES (253.62 ± 61.7 vs. 9.23 ± 5.8 and 23.95 ± 5.3 *ρ*g/mL), fractalkine (30.07 ± 3.1 vs. 3.91 ± 5.5 and 7.22 ± 5.8 *ρ*g/mL), IL-6 (45.27 ± 9.7 vs. 5.88 ± 5.3 and 14.80 ± 4.2 *ρ*g/mL), IL-7 (12.26 ± 1.8 vs. 1.18 ± 1.7 and 1.58 ± 2.2 *ρ*g/mL), IL-8 (707.73 ± 205.4 vs. 59.94 ± 30.8 and 110.29 ± 20.0 *ρ*g/mL), MCP-1 (28.01 ±7.3 vs. 2.38 ± 1.4 and 6.31 ± 1.1 *ρ*g/mL), and IP-10 (146.15 ± 45.1 vs. 2.38 ± 3.4 and 7.53 ± 10.6 *ρ*g/mL) (Fig.5). IFNA2 (8.15 ± 2.6 *ρ*g/mL), GM-CSF (10.19 ±3.1 *ρ*g/mL), and MIP-1*α* (18.74 ± 4.7 *ρ*g/mL) were only detected in the c.a. IKK*β* condition.

## Discussion

Skeletal muscle resident pericytes have known roles in muscle repair and regeneration. Previous work in our laboratory demonstrated that pericytes activate NF-*κ*B in response to muscle damage in humans. In support of previous work, our first main finding provides further in vitro evidence for pericytes as a source of NF-*κ*B activation following muscle damage. Second, through genetic manipulation, we showed that NF-*κ*B activation in pericytes enhanced the proliferation of cocultured endothelial cells. Finally, we identified several paracrine-signaling molecules that may mediate the crosstalk between pericytes and endothelial cells.

### Pericytes activate NF-*κ*B in response to muscle damage

Previous studies have documented the importance of NF-*κ*B activation in skeletal muscle tissue for the regulation of myogenesis (Guttridge et al. 1999, 2000; Peterson et al. 2011) as well as in muscle injury (Mourkioti et al. 2006) and disease (Cai et al. 2004). Hyldahl et al. (2011) demonstrated pericyte NF-*κ*B activation in muscle injury and regeneration in humans. In this study, we provide further evidence for pericytes as a source of NF-*κ*B activation. Our in vitro model of acute muscle injury allowed us to quantify nuclear NF-*κ*B binding activity and suggests that pericytes may potentially activate NF-*κ*B to a greater extent than muscle cells, although only a trend for increased pericyte NF-*κ*B activation was achieved in this study. Nevertheless, the data support our hypothesis that pericytes are key mediators of the inflammatory response during skeletal muscle regeneration. This model also allowed us to examine the crosstalk mechanisms that promote NF-*κ*B activation in pericytes.

At early time points following scratch-injury to muscle cells, MCP-1 was secreted by muscle cells, although no statistical difference between scratch-injured and control cultures was detected. Other studies have observed increased cytokine secretion from C_2_C_12_ cells using various models of muscle stress. Peterson and Pizza (2009) showed that C_2_C_12_ cells secreted MCP-1 in response to in vitro mechanical strain. Using an in vitro exercise model, Scheler et al. (2013) observed gene enrichment of NF-*κ*B related genes, including the CCL2 gene that encodes the MCP-1 protein, as well as CCL5 and CXCL1 genes, which encode RANTES and growth regulated oncogene (GRO) proteins, respectively. They also showed increased secretion of the MCP-1 protein (Scheler et al. 2013). In a human study, Catoire et al. (2014) found increased gene expression and protein secretion of MCP-1 in muscle biopsies and plasma, respectively, again highlighting the role of this cytokine in the muscle tissue response to stress; however, the cellular sources of MCP-1 were not determined in that study. In this study, we utilized a coculture with pericytes, and our finding of no change in MCP-1 secretion in response to acute injury, which is in contrast to previous studies, may indicate that pericytes modulated the inflammatory response in muscle cells, and thereby attenuated significant MCP-1 secretion from muscle cells. In the current study, we also showed that pericytes could secrete MCP-1, and further, that they may secrete greater quantities of MCP-1 than muscle cells. This provides further evidence that pericytes are important components of muscle cell crosstalk. Still, other cytokines should be investigated using this model to fully understand the cell crosstalk between pericytes and muscle cells.

### Pericyte NF-*κ*B activation enhances endothelial cell proliferation

The downstream effects of NF-*κ*B activation on cells in the muscle tissue environment are mostly unknown. Due to the close physical proximity between pericytes and endothelial cells, as well as the importance of a healthy microvasculature to promote muscle repair, we investigated the effects of pericyte NF-*κ*B activation on endothelial cells. We showed that NF-*κ*B activation in pericytes enhanced endothelial cell proliferation. Endothelial cell proliferation is one component of angiogenesis, indicating that pericyte NF-*κ*B activation may be important for the promotion of angiogenesis. Hyldahl et al. (2013) demonstrated that increased pericyte NF-*κ*B activation enhanced myoblast proliferation in coculture while also inhibiting pericyte differentiation into myotubes. These two studies show that acute NF-*κ*B activation can enhance cellular proliferation in skeletal muscle tissue. This is in contrast to studies showing that chronic NF-*κ*B activation in disease can lead to cachexia or muscle wasting (Cai et al. 2004), and thus demonstrates the paradoxical effects of inflammation. While acute inflammation may be necessary for tissue repair and vascular remodeling (Silvestre et al. 2008), chronic inflammation has negative effects (Khatami 2011). Importantly, if pericytes are mediators of the inflammatory response, this highlights the importance of a healthy pericyte population that can respond to damage and execute appropriate inflammatory signaling.

### Pericyte NF-*κ*B activation enhances inflammatory and angiogenic crosstalk

In order to elucidate cytokines that may be involved in the paracrine signaling that mediates the proliferative response in endothelial cells, we used an unbiased technique to screen the conditioned media of endothelial cell/pericyte cocultures and pericyte monocultures. Using this approach, we saw differential concentrations of several cytokines in the various NF-*κ*B activation conditions. All these cytokines are known gene targets of NF-*κ*B; and therefore, further supports the efficacy of our transfection. The cytokines that were present in the highest concentration in our cultures were IL-8, IP-10, and RANTES, and with the addition of IL-7, this group of cytokines also had the greatest fold change in the enhanced NF-*κ*B activation condition compared to the control condition. IL-8 is a proangiogenic cytokine in the CXC family, and it signals through the CXCR2 receptor to directly affect angiogenesis (Koch et al. 1992; Strieter et al. 1995). In a model of thyroid cancer, Bauerle and colleagues (Bauerle et al. 2014) showed that NF-*κ*B regulated the expression of IL-8, which enhanced tumor cell growth in vivo and endothelial cell tube formation in vitro. IP-10 is another member of the CXC family of cytokines, but contrary to IL-8, it is angiostatic and signals through the CXCR3 receptor (Romagnani et al. 2001; Mehrad et al. 2007). In addition to the concentration of signaling molecules, receptor concentration on endothelial cells are also important in the balance between angiogenesis and angiostasis. Although our study was not designed to investigate receptor density, it is possible that there are more CXCR2 receptors than CXCR3 receptors on our HMVECs. Furthermore, some levels of angiostatic cytokines are likely necessary to balance angiogenic signals and prevent uncontrolled cell growth.

MCP-1 and RANTES, members of the CC cytokine family, were also increased in the coculture condition of HMVECs and pericytes with enhanced NF-*κ*B activation. Many cell types may secrete MCP-1 following damage, and this study shows that pericytes secrete MCP-1 following NF-*κ*B activation. MCP-1 is mainly known for its role in leukocyte chemoattraction; however, previous studies have also shown a role for MCP-1 in angiogenesis (Salcedo et al. 2000; Ma et al. 2007). Ma et al. (2007) showed that MCP-1 mediated angiogenesis through the recruitment of mural cells, which provides a link between inflammatory signaling molecules and angiogenesis. The role of RANTES in angiogenesis is more controversial (Suffee et al. 2011). It can signal through multiple surface receptors where some studies have observed a proangiogenic effect (Westerweel et al. 2008) and others have demonstrated antiangiogenic effects (Barcelos et al. 2009). Importantly, the candidate cytokines that were identified in this study as NF-*κ*B driven mediators of pericyte-endothelial cell crosstalk are known inflammatory signaling molecules. Therefore, this study provides evidence for the link between inflammation and angiogenesis.

## Limitations

There are several recognized limitations to this study. First, we did not directly quantify muscle damage from our in vitro scrape injury model. Next, we did not extend our time series to 48 h in our pericyte/C_2_C_12_ cocultures, which may have revealed significant differences in pericyte NF-*κ*B activation between injured and control conditions. Another limitation of this study is a lack of a secondary measure of endothelial cell proliferation. The impact of the endothelial cell proliferation results is limited by a lack of a statistically significant difference between endothelial cell numbers in the control condition compared to either increased or decreased NF-*κ*B activation conditions. Future studies should follow up on the candidate cytokines identified in this study to identify which cytokines promote proliferation. Finally, these findings would be strengthened by validation in an in vivo model and investigation into the reciprocal effects of endothelial cells on pericytes.

## Conclusions

Skeletal muscle is a complex tissue with a high regenerative capacity. However, debilitating diseases and injuries affect skeletal muscle function and regeneration. In order to target and optimize therapies, the cellular and molecular mechanisms of muscle regeneration must be understood, including the crosstalk between different cell types. We have provided further evidence that pericyte NF-*κ*B activation may be an important component of the skeletal muscle inflammatory response to injury. We have also shown that pericyte NF-*κ*B activation can influence endothelial cell proliferation, which occurs through paracrine signaling with the candidate inflammatory cytokines that were identified in our screen. As components of the microvasculature, pericytes and endothelial cells are essential elements of a healthy skeletal muscle tissue; and therefore, this study may also have implications for musculoskeletal diseases as well as vascular diseases, including peripheral artery disease.
